# Association between psychological symptoms and illegal driving behaviors in a sample of Chinese private car drivers

**DOI:** 10.3389/fpsyt.2022.984860

**Published:** 2022-10-13

**Authors:** Hongguang Chen, Hui Li, Changqin Pu, Hubo Xu, Tingwei Wang, Ling Du, Xiuxiu Liu, Shunfei Li, Mengqian Li

**Affiliations:** ^1^Key Laboratory of Mental Health, Peking University Sixth Hospital (Institute of Mental Health), Ministry of Health (Peking University), National Clinical Research Center for Mental Disorders (Peking University Sixth Hospital), Beijing, China; ^2^Department of Psychosomatic Medicine, The First Affiliated Hospital of Nanchang University, Nanchang, China; ^3^Health Science Center, Peking University, Beijing, China; ^4^Yulin Center for Disease Control and Prevention, Yulin, China; ^5^The Fourth Hospital of Yulin, Yulin, China; ^6^The Second Hospital of Yulin, Yulin, China; ^7^Chinese People's Liberation Army of China (PLA) General Hospital, Beijing, China

**Keywords:** psychological symptoms, private car drivers, traffic violations, traffic accidents, SCL-90

## Abstract

**Background:**

Findings on the associations between psychological symptoms and driving behaviors in private car drivers are inadequate.

**Method:**

The study consisted of 3,115 private car drivers in Yulin, China. The measurements included socio-demographic data, traffic violations, accidents, and Symptom Checklist-90 (SCL-90). In addition, an ordered logistic regression model was employed to examine the association between each psychological symptom and risky driving behaviors.

**Results:**

The overall prevalence rate of any self-reported psychological symptom was 10.24%, with 9.22% for males and 11.49% for females. Among them, obsessive-compulsive, interpersonal sensitivity, additional items, hostility, and depression were the five most common psychological symptoms, with prevalence rates of 7.90, 6.29, 6.00, 5.91, and 5.62%, respectively. Any psychological symptom factor was associated with a higher risk of traffic violations and accidents. However, the intensity of the correlations varied, with obsessive-compulsive symptoms the strongest in general traffic violations and anxiety symptoms in traffic accidents. All psychological symptoms except phobic anxiety and paranoid ideation contributed to a higher risk of failing the driver's license test.

**Conclusions:**

The prevalence rate of psychological symptoms was high in private car drivers. This study calls for an urgent need to establish a pilot tertiary prevention strategy to reduce risky driving behaviors through psychological symptom screening and interventions among private car drivers.

## Introduction

With the development and progress of the economy and society, traveling by car has become a common choice. However, the significant annual traffic accidents lead to a severe loss of life and property. According to the statistics from WHO, about 1.35 million people die from road traffic accidents every year, and road traffic accidents have become the leading cause of death among children and adolescents aged 5–29 (1). In China, according to the statistics of the Ministry of Public Security, the number of motor vehicles in China reached 395 million in 2021, including 302 million cars, and the number of motor vehicle drivers reached 481 million, including 444 million car drivers (1–3). The China Statistical Yearbook showed that the total number of traffic accidents in 2020 was 2,44, 674, with 61,703 people killed, 2,50,723 injured, and 1.31 billion RMB in direct property losses. In recent years, studies on the prevalence of psychological symptoms and associated correlators in motor vehicle drivers received some coverage (4, 5). For example, it is reported that occupational strain, neuroticism, and psychological symptoms are all positively correlated in metro drivers (6). Also, the study reported that bus drivers were significantly more extroverted, psychotic, and neurotic than the general population; and they were more likely to suffer from somatization and phobia (5). Similarly, some studies have reported the relationship between psychological status and traffic violations, especially for professional drivers. Data showed that bus drivers' traffic violations were mainly affected by specific socio-demographic characteristics, personality traits, and mental health, which increase the risk of traffic violations (7). Multiple personality traits, such as oppositional and negative affectivity, were also influential in predicting speeding and risky driving behaviors (8–10). Studies have also attempted to use the psychological state as a predictor of traffic violations and noted that drivers suffering from depression, anxiety, and neuroticism exhibit more risky driving behaviors (11, 12). However, most current studies come from western countries and few from low-and middle-income countries. In addition, previous studies mainly focus on professional drivers, including bus drivers, taxi drivers, subway drivers, and train drivers, and seldom involve the most significant number of private car drivers. In addition, previous studies mainly focused on the prevalence of one specific psychological symptom of the drivers but less involved a wide range of psychological symptoms or in specific traffic violations and traffic accidents simultaneously (13–15). Hence, in a Chinese sample, this study was designed to explore the prevalence of psychological symptoms in private car drivers and their associations with risky traffic behaviors and related accidents. At the same time, the relationship between psychological symptoms and the number of times the private car drivers passed the test was also analyzed, providing the basis for early identification and intervention.

## Method

### Participants and procedure

A cross-sectional study was conducted to assess the psychological status of private car drivers in Yulin, a city located in the north of Shaanxi Province, P. R. China, which covers an area of 43,578 km^2^ and has jurisdiction over one district and eleven counties. The number of motor vehicles in the city has reached 1.028 million, with 1.244 million drivers. In China, private car drivers must complete a physical exam at an authorized hospital to prove their physical qualifications to drive. All drivers who passed the physical examination were mobilized to complete a questionnaire by scanning the Quick Response code (QR code) from January 1, 2021, to July 31, 2021. In addition, all respondents obtained electronic informed written consent before data collection. The Research Ethics Board approved the research at the First Affiliated Hospital of Nanchang University (2021-112).

### Measurements

#### Demographics and driving information

Demographic information was collected, including gender, age, education, household registration, residency, marital status, family income, self-reported physical illness, driving experience, and the frequencies that passed the driver's license test (DLT).

#### Traffic violations and traffic accident information

According to the illegal acts of the Tort Law of the People's Republic of China, the main illegal items were listed in the questionnaire, detailed in A1–A16 (see Table 1). In addition, preliminary traffic accident information was collected to explore further the relationship between PDs and traffic accidents, detailed in B1–B8.

**Table 1:** List for main traffic violations and traffic accident items.

**Traffic violations (A)**	**Traffic accidents (B)**
A1 drunk driving	B1 active rear-end collision accident
A2 driving unlicensed motor vehicles	B2 passive rear-end collision accident
A3 violation of traffic control	B3 head-on collision accident
A4 reversing or retrograde on highways and expressways	B4 motor vehicle scratch accident
A5 speeding on expressways	B5 scraping with pedestrians
A6 violates the traffic light instructions	B6 scratches with bicycles
A7 intentionally obstructs or defaces the license plate of a motor vehicle	B7 scrapes with other vehicles
A8 receiving phone calls and watching TV during driving	B8 others
A9 failing to stop, slow down or avoid pedestrians when passing through a crosswalk	
A10 failing to use seat belts	
A11 without a vehicle driving license	
A12 without a driver's license	
A13 overloaded or overcrowded	
A14 occupies the emergency lane	
A15 line-pressing driving	
A16 others	

#### Psychological status

Self-reported psychological symptoms were assessed using the Symptom Checklist-90 (SCL-90). Previous studies have verified the reliability and validity of the Chinese version of the SCL-90 (16, 17). The inventory contains 90 questions that evaluate 10 primary symptom factors in the last week: somatization, obsessive-compulsive, interpersonal sensitivity, depression, anxiety, hostility, phobic anxiety, paranoid ideation, psychoticism, and additional items (e.g., appetite and sleep). Each of the 10 symptom factors contains 6–13 items. All items were graded on a 5-point Likert scale, ranging from “1 = not at all” to “5 = extremely,” with a higher score indicating more frequency and intensity of psychological symptoms. The mean score of each factor was used as the indicator to evaluate the mental health status. When a factor score was ≥2, indicating mental health problems in that factor (18).

### Statistical analysis

Descriptive statistics were used to characterize the sample. Continuous variables are presented as the mean ± SD (Standard deviation) and median (IQR, Interquartile range), whereas categorical variables are as numbers (*n*) and percentages (%). Previous studies have reported differences in the prevalence of psychological symptoms between males and females (19, 20), so Chi-squared tests were used to examine the statistical differences in prevalence rates of self-reported psychological symptoms by gender. Kruskal-Wallis rank test was used to compare the prevalence rate of each psychological symptom in traffic violations, traffic accidents, and DLT, and any psychological symptom at different times of each traffic violation and traffic accident. A tetrachoric correlation was used to examine the correlation between each psychological symptom. Ordered logistic regression models were fitted to examine the association between each psychological symptom and traffic violations/traffic accidents, adjusted for potential confounders. In addition, we further analyze the associations between each psychological symptom and frequencies of passing the DLT using the ordered logistic regression models. Statistical significance was considered a two-tailed *P*-value < 0.05. All analyses were performed under the SPSS v19 (SPSS Inc., USA).

## Results

### Demographic characteristics and driving information

During the study, the response rate of the subjects was 75.42%. After excluding cases with incomplete information, 3,115 subjects with an average age of 33.67 years (SD 9.64) were included in the analysis, with 1,714 male (55.02%, see Table 2) and 1,401 female drivers (44.98%). As a result, there were 1,269 (40.74%), 923 (29.63%), and 923 (29.63%) drivers with high school education or below, college degree, and bachelor's degree or above, respectively. In addition, among all the subjects, 2,031 (65.20%) registered as agricultural residents, 2,234 (71.72%) living in the urban areas, 1,866 (59.90%) had an average monthly household income of less than $5,000 RMB, 3,002 (96.37%) had no self-reported histories of somatic diseases, and 615 (19.74%) passed the DLT more than once.

**Table 2:** Demographic characteristics of all 3,115 investigated private car drivers.

**Variables**	**Group**	***N* (%)**
Age (years), mean (SD)		33.67 (9.64)
Gender	Male	1,714 (55.02)
	Female	1,401 (44.98)
Educational level	High school or below	1,269 (40.74)
	College degree	923 (29.63)
	Bachelor's degree or above	923 (29.63)
Household registration	Agricultural Hukou (residency)	2,031 (65.20)
	Non-agricultural Hukou (residency)	1,084 (34.80)
Residence	Urban	2,234 (71.72)
	Rural	881 (28.28)
Marital status	Unmarried	919 (29.50)
	First marriage	1,999 (64.17)
	Remarriage	124 (3.98)
	Divorced or widowed	73 (2.34)
Average monthly household income	≤5,000 RMB	1,866 (59.90)
	>5,000 RMB	1,249 (40.10)
Self-reported somatic disease	No	3,002 (96.37)
	Yes	113 (3.63)
Years of driving experience (years), median (IQR)		5 (2.00–8.91)
Frequencies before passing the DLT	1	2,500 (80.26)
	2	515 (16.53)
	Three or more	100 (3.21)

### Prevalence of self-reported psychological symptoms

Among private drivers, the prevalence rate of any self-reported psychological symptom was 10.24%, with 9.22% for males and 11.49% for females. As for the prevalence of symptoms, obsessive-compulsive, interpersonal sensitivity, additional items, hostility, and depression were the five most common psychological syndromes, accounting for 7.90, 6.29, 6.00, 5.91, and 5.62% (see Table 3), respectively. Meanwhile, the prevalence rates of somatization, anxiety, phobic anxiety, paranoid ideation, and psychoticism were 4.94, 4.88, 4.59, 5.33, and 4.82%, respectively.

**Table 3:** Prevalence of self-reported psychological symptoms and stratified by gender^*^.

	***N***	**Prevalence (95%CI)**	**Male**		**Female**		***P*-value**
			***N***	**Prevalence (95% CI)**	***N***	**Prevalence (95% CI)**	
Somatization, i ≥ 2	154	4.94 (4.24–5.76)	68	3.97 (3.19–4.92)	86	6.14 (4.90–7.66)	0.005
Obsessive-compulsive, i ≥ 2	246	7.90 (7.00–8.90)	112	6.53 (5.53–7.71)	134	9.56 (8.02–11.37)	0.002
Interpersonal sensitivity, i ≥ 2	196	6.29 (5.49–7.20)	91	5.31 (4.36–6.45)	105	7.49 (6.31–8.88)	0.012
Depression, i ≥ 2	175	5.62 (4.86–6.48)	78	4.55 (3.65–5.67)	97	6.92 (5.73–8.35)	0.004
Anxiety, i ≥ 2	152	4.88 (4.18–5.69)	70	4.08 (3.24–5.13)	82	5.85 (4.60–7.41)	0.023
Hostility, i ≥ 2	184	5.91 (5.13–6.79)	83	4.84 (3.86–6.05)	101	7.21 (5.98–8.67)	0.005
Phobic anxiety, I ≥ 2	143	4.59 (3.91–5.38)	68	3.97 (3.10–5.06)	75	5.35 (4.09–6.98)	0.066
Paranoid ideation, i ≥ 2	166	5.33 (4.59–6.18)	81	4.73 (3.79–5.88)	85	6.07 (4.81–7.63)	0.097
Psychoticism, i ≥ 2	150	4.82 (4.12–5.63)	72	4.2 (3.34–5.27)	78	5.57 (4.40–7.02)	0.076
Additional items, i ≥ 2	187	6.00 (5.22–6.89)	91	5.31 (4.32–6.51)	96	6.85 (5.48–8.54)	0.071
Any of the above items	319	10.24 (9.28–11.29)	158	9.22 (7.94–10.68)	161	11.49 (9.91–13.29)	0.037

^*^Based on investigated sample size and the result of the study, *post-hoc* power estimation showed that a sample size of 3,115 could achieve 99.9% power to detect a difference of 0.03 (under the null hypothesis is 0.13) using an exact two-sided test with a significance level of 0.05.

### Prevalence of each psychological symptom among drivers with different types of traffic violations

The numbers of drivers with any symptoms who experienced no traffic violation, one traffic violation, and more than one type of traffic violation were 211 (7.90%, see Table 4), 63 (22.74%), and 45 (26.95%), respectively. All 10 symptoms were statistically related to traffic violations. For private car drivers with one type of traffic violation, the proportion of obsessive-compulsive was highest (18.05%), followed by interpersonal sensitivity (13.36%) and additional items (10.83). Among drivers with multiple types of traffic violations, the proportion of obsessive-compulsive was again the highest (20.36%), followed by interpersonal sensitivity (17.96%) and hostility (16.77%).

**Table 4:** Prevalence of each psychological symptom among drivers with different types of traffic violations.

	**No traffic violation**	**One traffic violation**	**More than one type**	***P*-value**
	***n* (%)**	***n* (%)**	***n* (%)**	
Somatization, i ≥ 2	107 (4.01)	24 (8.66)	23 (13.77)	<0.001
Obsessive-compulsive, i ≥ 2	162 (6.07)	50 (18.05)	34 (20.36)	<0.001
Interpersonal sensitivity, i ≥ 2	129 (4.83)	37 (13.36)	30 (17.96)	<0.001
Depression, i ≥ 2	121 (4.53)	27 (9.75)	27 (16.17)	<0.001
Anxiety, i ≥ 2	104 (3.89)	26 (9.39)	22 (13.17)	<0.001
Hostility, i ≥ 2	127 (4.75)	29 (10.47)	28 (16.77)	<0.001
Phobic anxiety, i ≥ 2	104 (3.89)	19 (6.86)	20 (11.98)	<0.001
Paranoid ideation, i ≥ 2	114 (4.27)	24 (8.66)	28 (16.77)	<0.001
Psychoticism, i ≥ 2	108 (4.04)	21 (7.58)	21 (12.57)	<0.001
Additional items, i ≥ 2	129 (4.83)	30 (10.83)	28 (16.77)	<0.001
Any of the above items	211 (7.90)	63 (22.74)	45 (26.95)	<0.001

### Prevalence of each psychological symptom among drivers with different types of traffic accidents

For private car drivers with no traffic accident, one traffic accident, and more than one type of traffic accident, the prevalence rates of individuals suffering from any psychological symptom were 9.47, 15.97, and 41.18% (see Table 5), respectively. All symptoms were identified as related to traffic accidents. Among private car drivers with one type of accident, the proportion of individuals with obsessive-compulsive was the highest (11.76%), followed by interpersonal sensitivity (9.24%) and hostility (9.24%). In drivers with multiple traffic accidents, obsessive-compulsive and anxiety were the highest, with each accounting for 29.41%.

**Table 5:** Prevalence of each psychological symptom with drivers with different types of traffic accidents.

	**No traffic accident**	**One traffic accident**	**More than one type**	***P*-value**
	***n* (%)**	***n* (%)**	***n* (%)**	
Somatization, i ≥ 2	138 (4.69)	4 (3.36)	12 (23.53)	<0.001
Obsessive-compulsive,i ≥ 2	217 (7.37)	14 (11.76)	15 (29.41)	<0.001
Interpersonal sensitivity, i ≥ 2	171 (5.81)	11 (9.24)	14 (27.45)	<0.001
Depression, i ≥ 2	153 (5.20)	8 (6.72)	14 (27.45)	<0.001
Anxiety, i ≥ 2	130 (4.41)	7 (5.88)	15 (29.41)	<0.001
Hostility, i ≥ 2	159 (5.40)	11 (9.24)	14 (27.45)	<0.001
Phobic anxiety, i ≥ 2	126 (4.28)	5 (4.20)	12 (23.53)	<0.001
Paranoid ideation, i ≥ 2	145 (4.92)	7 (5.88)	14 (27.45)	<0.001
Psychoticism, i ≥ 2	134 (4.55)	3 (2.52)	13 (25.49)	<0.001
Additional items, i ≥ 2	163 (5.53)	10 (8.40)	14 (27.45)	<0.001
Any of the above items	279 (9.47)	19 (15.97)	21 (41.18)	<0.001

### Prevalence of each psychological symptom among drivers with different frequencies of passing the driver's license test

All symptoms were statistically associated with times passed the DLT. Among those who failed the DLT for the first time, 15.28% (data not shown) had any psychological symptoms. For drivers who passed the DLT with second or more attempts, obsessive-compulsive appeared the highest, accounting for 12.36%, followed by interpersonal sensitivity (9.43%). Detailed information for the correlations between each psychological factor and frequencies of passing the DLT is shown in Table 6.

**Table 6:** Prevalence of each psychological symptom among drivers with different frequencies of passing the DLT.

	**Pass on the first attempt**	**Pass at the second attempt**	**Pass at three times and above**	***P*-value**
	***n* (%)**	***n* (%)**	***n* (%)**	
Somatization, i ≥ 2	107 (4.28)	36 (6.99)	11 (11.00)	<0.001
Obsessive-compulsive,i ≥ 2	170 (6.80)	57 (11.07)	19 (19.00)	<0.001
Interpersonal sensitivity, i ≥ 2	138 (5.52)	42 (8.16)	16 (16.00)	<0.001
Depression, i ≥ 2	123 (4.92)	38 (7.38)	14 (14.00)	<0.001
Anxiety, i ≥ 2	108 (4.32)	33 (6.41)	11 (11.00)	<0.001
Hostility, i ≥ 2	132 (5.28)	39 (7.57)	13 (13.00)	<0.001
Phobic anxiety, i ≥ 2	105 (4.20)	27 (5.24)	11 (11.00)	0.005
Paranoid ideation, i ≥ 2	122 (4.88)	32 (6.21)	12 (12.00)	0.004
Psychoticism, i ≥ 2	108 (4.32)	30 (5.83)	12 (12.00)	0.001
Additional items, i ≥ 2	135 (5.40)	42 (8.16)	10 (10.00)	0.003
Any of the above items	225 (9.00)	72 (13.98)	22 (22.00)	<0.001

### Prevalence of any psychological symptom among drivers with different times of each traffic violation/traffic accident

For private car drivers, the number of past year's self-reported traffic violations and traffic accidents was 444 and 170, respectively (see Table 7). Among all types of traffic violations and traffic accidents, the proportion of 'other kind of traffic violations' was the highest in traffic violations, accounting for 29.95%, and motor vehicle scratch accident was the highest in traffic accidents (45.88%).

**Table 7:** Prevalence of any psychological symptom among drivers with different frequencies of each traffic violation/traffic accident.

**Variables**	***N***	**%**	**Any psychological symptom**			***P*-value**
			**None**	**One time**	**Two times and above**	
**Traffic violations (A)**	**444**	**–**				<0.001
A1 drunk driving	41	9.23	303 (9.86)	13 (52.00)	3 (18.75)	<0.001
A2 driving unlicensed motor vehicles	25	5.63	306 (9.90)	9 (56.25)	4 (44.44)	<0.001
A3 violation of traffic control	26	5.86	307 (9.94)	6 (42.86)	6 (50.00)	<0.001
A4 reversing or retrograde on highways and expressways	30	6.76	301 (9.76)	11 (68.75)	7 (50.00)	<0.001
A5 speeding on expressways	100	22.52	290 (9.62)	19 (26.03)	10 (37.04)	<0.001
A6 violates the traffic light instructions	131	29.50	290 (9.72)	23 (21.50)	6 (25.00)	<0.001
A7 intentionally obstructs or defaces the license plate of a motor vehicle	29	6.53	304 (9.85)	11 (64.71)	4 (33.33)	<0.001
A8 receiving phone calls and watching TV during driving	50	11.26	292 (9.53)	21 (58.33)	6 (42.86)	<0.001
A9 failing to stop, slow down or avoid pedestrians when passing through a crosswalk	110	24.77	288 (9.58)	23 (26.14)	8 (36.36)	<0.001
A10 failing to use seat belts	82	18.47	296 (9.76)	15 (21.74)	8 (61.54)	<0.001
A11 without a vehicle driving license	38	8.56	302 (9.81)	11 (40.74)	6 (54.55)	<0.001
A12 without a driver's license	24	5.41	306 (9.90)	7 (50.00)	6 (60.00)	<0.001
A13 overloaded or overcrowded	27	6.08	306 (9.91)	8 (47.06)	5 (50.00)	<0.001
A14 occupies the emergency lane	32	7.21	301 (9.76)	11 (52.38)	7 (63.64)	<0.001
A15 line-pressing driving	85	19.14	292 (9.64)	20 (27.40)	7 (58.33)	<0.001
A16 others	133	29.95	284 (9.52)	21 (22.83)	14 (34.15)	<0.001
**Traffic accidents (B)**	**170**	**–**				
B1 active rear-end collision accident	46	27.06	301 (9.81)	12 (33.33)	6 (60.00)	<0.001
B2 passive rear-end collision accident	59	34.71	300 (9.82)	12 (26.09)	7 (53.85)	<0.001
B3 head-on collision accident	28	16.47	303 (9.82)	10 (55.56)	6 (60.00)	<0.001
B4 motor vehicle scratch accident	78	45.88	297 (9.78)	15 (23.44)	7 (50.00)	<0.001
B5 scraping with pedestrians	35	20.59	303 (9.84)	9 (39.13)	7 (58.33)	<0.001
B6 scratches with bicycles	26	15.29	305 (9.87)	7 (50.00)	7 (58.33)	<0.001
B7 scrapes with other vehicles	40	23.53	303 (9.85)	9 (33.33)	7 (53.85)	<0.001
B8 others	33	19.41	305 (9.90)	8 (36.36)	6 (54.55)	<0.001

For drivers with a one-time traffic violation, “reversing or retrograde on highways and expressways” was the highest (68.75%) among individuals with any psychological symptom, followed by “intentionally obstructs or defaces the license plate of a motor vehicle” (64.71%) and receiving phone calls and watching TV during driving (58.33%). For drivers with two times and above traffic violations, “occupies the emergency lane” (63.64%) was the highest, followed by “failing to use seat belts” (61.54%) and “without a driver's license” (60%) among individuals with any psychological symptom. Finally, for drivers with one or two traffic violations, “head-on collision accidents” (one time, 55.56%; two or more times, 60%) were the highest proportion of individuals with any psychological symptoms.

### Impact of self-reported psychological symptoms on traffic violations/traffic accidents/frequencies of passing the DLT

After adjusting for factors such as age, gender, educational level, household registration, residence, marriage, average monthly household income, self-reported somatic disease, years of driving experience, and the frequencies of passing the DLT, results from ordered logistic regression indicated that all types of self-reported psychological symptoms were associated with traffic violations. Of which, obsessive-compulsive had the strongest association (OR, 3.54, 95%CI: 2.66–4.70, see Table 8), followed by interpersonal sensitivity (OR, 3.41, 95%CI: 2.49–4.66), and paranoid ideation (OR, 3.12, 95%CI: 2.21–4.40).

**Table 8:** Associations between each psychological symptom and traffic violation/traffic accident/frequencies of passing the driver's license's test examined by ordered logistic regression.

	**Traffic violation** ^**†**^		**Traffic accident** ^**†**^		**Times of passing the test** ^**‡**^	
	**cOR (95CI%)**	**aOR (95%CI)**	**cOR (95CI%)**	**aOR (95%CI)**	**cOR (95CI%)**	**aOR (95%CI)**
Somatization, i ≥ 2	2.92 (2.05–4.17)***	2.82 (1.97–4.04)***	2.23 (1.30–3.84)**	2.07 (1.20–3.57)**	1.89 (1.33–2.70)***	1.74 (1.21–2.49)**
Obsessive-compulsive,i ≥ 2	3.56 (2.69–4.72)***	3.54 (2.66–4.70)***	2.66 (1.75–4.07)***	2.51 (1.64–3.85)***	2.00 (1.50–2.65)***	1.90 (1.42–2.54)***
Interpersonal sensitivity, i ≥ 2	3.53 (2.59–4.81)***	3.41 (2.49–4.66)***	2.91 (1.85–4.57)***	2.65 (1.67–4.18)***	1.86 (1.35–2.56)***	1.75 (1.27–2.43)**
Depression, i ≥ 2	3.02 (2.16–4.22)***	2.96 (2.11–4.16)***	2.85 (1.77–4.59)***	2.66 (1.65–4.31)***	1.86 (1.33–2.60)***	1.75 (1.24–2.46)**
Anxiety, i ≥ 2	3.04 (2.13–4.32)***	3.04 (2.13–4.34)***	3.44 (2.12–5.57)***	3.19 (1.96–5.19)***	1.76 (1.23–2.52)**	1.63 (1.13–2.36)**
Hostility, i ≥ 2	3.04 (2.20–4.22)***	3.04 (2.19–4.23)***	3.15 (2.00–4.95)***	2.97 (1.88–4.69)***	1.71 (1.22–2.38)**	1.62 (1.16–2.28)**
Phobic anxiety, i ≥ 2	2.47 (1.69–3.61)***	2.48 (1.69–3.65)***	2.63 (1.54–4.48) **	2.42 (1.41–4.15)**	1.57 (1.07–2.30)*	1.40 (0.95–2.06)
Paranoid ideation, i ≥ 2	3.14 (2.23–4.42)***	3.12 (2.21–4.40)***	2.87 (1.77–4.68)***	2.67 (1.63–4.36)***	1.56 (1.09–2.23)*	1.41 (0.98–2.03)
Psychoticism, i ≥ 2	2.56 (1.77–3.71)***	2.56 (1.77–3.72)***	2.32 (1.35–4.01)**	2.13 (1.23–3.70)**	1.70 (1.18–2.45)**	1.56 (1.07–2.26)*
Additional items, i ≥ 2	3.04 (2.20–4.21)***	3.00 (2.17–4.17)***	2.93 (1.85–4.64)***	2.79 (1.75–4.43)***	1.63 (1.17–2.27)**	1.50 (1.07–2.10)*
Any of the above items	3.73 (2.89–4.81)***	3.46 (2.67–4.50)***	3.04 (2.09–4.43)***	2.66 (1.81–3.91)***	1.87 (1.45–2.42)***	1.79 (1.38–2.33)***

^***^P < 0.001, ^**^P < 0.01, ^*^P < 0.05;^†^Adjusting for age, gender, educational level, household registration, residence, marriage, average monthly household income, self-reported somatic disease, years of driving experience, and the number of times passed the DLT, dependent variables were ordered by “No traffic violation,” “One traffic violation,” “More than one type and “No traffic accident,” “One traffic accident,” “More than one type” respectively;^‡^Adjusting for age, gender, educational level, household registration, residence, marriage, average monthly household income, self-reported of somatic disease; dependent variable was ordered by “Pass on the first attempt,” “Pass at the second attempt” and Pass at three times and above.”

As for traffic accidents, all types of self-reported psychological symptoms were associated with traffic accidents, with anxiety the strongest association (OR, 3.19, 95%CI: 1.96–5.19), followed by hostility (OR, 2.97, 95%CI: 1.88–4.69), and additional items (OR, 2.79, 95%CI: 1.75–4.43).

As for the number of frequencies passing the DLT, all types of self-reported psychological symptoms except phobic anxiety and paranoid ideation were associated with this indicator. Among them, obsessive-compulsive was the highest (OR, 1.90, 95%CI: 1.42–2.54), followed by interpersonal sensitivity (OR, 1.75, 95%CI: 1.27–2.43) and depression (OR, 1.75, 95%CI: 1.24–2.46).

## Discussion

Our paper is the first study to explore the prevalence of psychological symptoms and their impact on driving behavior in a sample of Chinese private vehicle drivers. The overall prevalence rate of any psychological symptom was 10.24%, higher than that of the general population in the absence of COVID-19 (19, 21), lower than that of the general public during the level I emergency response to COVID-19 and medical health workers (22–24). The difference in prevalence seemed to be related to the area, survey tool, survey period, and characteristics of the survey's target group. Previous studies have noted that younger drivers were more likely to experience psychological symptoms, and the prevalence of psychological symptoms was reported to rise during and after the post-COVID-19 era (25–27). Our findings showed that the a part of the highest prevalence ranked the same among the top five psychological symptoms-obsessive-compulsive followed by interpersonal sensitivity, additional items, hostility, and depression in the general population (24). However, the ranking order was not consistent with the survey results of bus drivers with high somatization and phobia scores (5). These inconsistencies were related to the difference in subjects, driving years, and gender composition. Not surprisingly, gender differences influenced the prevalence of psychological symptoms. Consistent with most previous research (28, 29), the prevalence rates of all psychological symptoms were higher in female drivers than in males.

In this study, all psychological symptoms seemed associated with traffic violations/accidents. Obsessive-compulsive and interpersonal sensitivity had the strongest correlation with traffic violations, while phobic anxiety had the weakest. Potential explanations include: (1) Drivers with interpersonal sensitivity or obsessive-compulsiveness often show excessive attention to other people or external influences, making it challenging to timely correct potentially risky driving behaviors. (2) Drivers with obsessive-compulsiveness or interpersonal sensitivity often exhibit concerns about traffic accidents and repeatedly-checking behaviors while driving (30, 31). The behavior mentioned above can further lead to inattentive driving and traffic violations. (3) Most obsessive-compulsive patients have psychiatric comorbidities, in which attention defense/hyperactivity disorder (ADHD) proved to be associated with risky driving behaviors (32, 33). Meanwhile, drivers with hostility often show difficulty in controlling their emotions and behaviors, making drivers more prone to aggressive or reckless behavior under stress and anger (34). Anxiety was identified as the most related factor among drivers with traffic accidents. Consistent with previous research findings, anxiety was associated with insomnia, loneliness, longer working hours, and a higher risk of traffic accidents. Our study noted that somatization was least associated with traffic accidents, which is inconsistent with previous findings (35). Wang et al. (5) claimed that somatization was a crucial marker for traffic accidents due to the driver's body's chronic vibration, leading to autonomic and vestibular organ dysfunction. The characteristics of target groups, driving time, and age distribution might be responsible for these differences (5, 15, 36). Our findings also indicated that psychological symptoms affect drivers in many ways, including the driving behavior after obtaining the driver's license and the success rate of passing the DLT for the first time. However, the influence of each psychological symptom on the driver's behavior was also different. It is worth noting that individuals with phobic anxiety and paranoid ideation were not statistically associated with the frequencies of passing the DLT in the first round, which might be related to the specific symptoms of the two symptoms with uncontrollable fear and anxiety about an unknown outcome, prompting them to repeat the exercises. Therefore, drivers who fail the DLT for the first time can be considered a critical group to undergo the early psychological screening program. This study has several limitations. First, recall bias related to self-reporting traffic violations and traffic accidents is not avoidable among these private car drivers. Second, this study is a non-probabilistic sampling survey, and the extrapolation of the results needs to be cautious. Finally, the SCL-90 checklist is a measurement for psychological symptoms screening rather than diagnosis.

## Conclusion

The prevalence rate of psychological symptoms was high in private car drivers. However, there are both overlaps and uniqueness among psychological symptoms and differences in the correlation and intensity between each psychological symptom and traffic violations/accidents. Both correlations between (a) obsessive-compulsive symptoms and general traffic violations and (b) anxiety symptoms and traffic accidents appeared the strongest, reminding us to pay more attention to these issues. Notably, failing the first-time driver's license test can be an early predictor for psychological screening since most psychological symptoms are associated with failing the driver's license test. Therefore, the following tertiary prevention strategies need to be in place to reduce risky driving behavior: (a) primary prevention, screening for psychological symptoms among those who failed their first driver's license test. (b) secondary prevention, screening for psychological symptoms among drivers with traffic violations, and offering necessary intervention. (c) screening and diagnosing psychological disorders and offering comprehensive intervention for those who have experienced traffic accidents.

## Data availability statement

The data that support the findings of this study are available from the corresponding author, upon reasonable request.

## Ethics statement

The studies involving human participants were reviewed and approved by the Research Ethics Board approved the research at the First Affiliated Hospital of Nanchang University (2021-112). The patients/participants provided their written informed consent to participate in this study.

## Author contributions

ML and SL had full access to all the data in the study and took responsibility for the integrity of the data and the accuracy of the data analysis and supervision. HC, SL, and ML: concept and design. TW, HC, and HL: acquisition, analysis, or interpretation of data. HC and CP: drafting of the manuscript. HC and HX: statistical analysis. SL and HC: obtained funding. TW, LD, and XL: administrative, technical, or material support. All authors: critical revision of the manuscript for important intellectual content.

## Funding

This study was supported by the National Major Science and Technology Project of China (Grant No. 2020YFC2003402) and the National Key R&D Program of China (Grant No. 2021YFF1201100).

## Conflict of interest

The authors declare that the research was conducted in the absence of any commercial or financial relationships that could be construed as a potential conflict of interest.

## Publisher's note

All claims expressed in this article are solely those of the authors and do not necessarily represent those of their affiliated organizations, or those of the publisher, the editors and the reviewers. Any product that may be evaluated in this article, or claim that may be made by its manufacturer, is not guaranteed or endorsed by the publisher.

## References

[1] Hogan AB Jewell BL Sherrard-Smith E Vesga JF Watson OJ Whittaker C. Potential impact of the Covid-19 pandemic on HIV, tuberculosis, and malaria in low-income and middle-income countries: a modeling study. Lancet Glob Health. (2020) 8:e1132–41. 10.1016/S2214-109X(20)30288-6 32673577 PMC7357988

[2] Jiang B Liang S Peng ZR Cong H Levy M Cheng Q. Transport and public health in China: the road to a healthy future. Lancet. (2017) 390:1781–91. 10.1016/S0140-6736(17)31958-X 29047445 PMC5704968

[3] Zhang Q Ge Y Qu W Zhang K Sun X. The traffic climate in China: the mediating effect of traffic safety climate between personality and dangerous driving behavior. Accid Anal Prev. (2018) 113:213–23. 10.1016/j.aap.2018.01.031 29428640

[4] van Vreden C Xia T Collie A Pritchard E Newnam S Lubman DI. The physical and mental health of Australian truck drivers: a national cross-sectional study. BMC Public Health. (2022) 22:464. 10.1186/s12889-022-12850-5 35260120 PMC8903653

[5] Wang X Wang K Huang K Wu X Huang W Yang L. The association between demographic characteristics, personality, and mental health of bus drivers in China: a structural equation model. Physiol Behav. (2021) 229:113247. 10.1016/j.physbeh.2020.113247 33197469

[6] He J Zhang Y Qin S Liu W. Assessment and discussion of correlation among psychological symptoms, occupational strain, and neurotic personality for metro drive. Front Psychol. (2022) 13:823682. 10.3389/fpsyg.2022.823682 35651565 PMC9149560

[7] Wang X Huang K Yang L. Effects of socio-demographic, personality and mental health factors on traffic violations in Chinese bus drivers. Psychol Health Med. (2019) 24:890–900. 10.1080/13548506.2019.1567928 30676085

[8] Scigala DK Zdankiewicz-Scigala E. The role in road traffic accident and anxiety as moderators attention biases in modified emotional stroop test. Front Psychol. (2019) 10:1575. 10.3389/fpsyg.2019.01575 31338054 PMC6629883

[9] Lucidi F Mallia L Lazuras L Violani C. Personality and attitudes as predictors of risky driving among older drivers. Accid Anal Prev. (2014) 72:318–24. 10.1016/j.aap.2014.07.022 25108900

[10] Shi X Zhang L. Effects of altruism and burnout on driving behavior of bus drivers. Accid Anal Prev. (2017) 102:110–5. 10.1016/j.aap.2017.02.025 28268203

[11] Ehsani JP Li K Simons-Morton BG Fox Tree-McGrath C Perlus JG O'Brien F. Conscientious personality and young drivers' crash risk. J Safety Res. (2015) 54:83–7. 10.1016/j.jsr.2015.06.015 26403906 PMC4583657

[12] Wang F Zhang J Wang S Li S Hou W. Analysis of driving behavior based on dynamic changes of personality states. Int J Environ Res Public Health. (2020) 17:1–17. 10.3390/ijerph17020430 31936406 PMC7013947

[13] Ge Y Qu W Jiang C Du F Sun X Zhang K. The effect of stress and personality on dangerous driving behavior among Chinese drivers. Accid Anal Prev. (2014) 73:34–40. 10.1016/j.aap.2014.07.024 25171523

[14] Hilton MF Staddon Z Sheridan J Whiteford HA. The impact of mental health symptoms on heavy goods vehicle drivers' performance. Accid Anal Prev. (2009) 41:453–61. 10.1016/j.aap.2009.01.012 19393792

[15] Huang YW Lin PC Wang J. The influence of bus and taxi drivers' public self-consciousness and social anxiety on aberrant driving behaviors. Accid Anal Prev. (2018) 117:145–53. 10.1016/j.aap.2018.04.014 29702332

[16] Tan H Lan XM Yu NL Yang XC. Reliability and validity assessment of the revised symptom checklist 90 for alopecia areata patients in China. J Dermatol. (2015) 42:975–80. 10.1111/1346-8138.12976 26072969

[17] Yu Y Wan C Huebner ES Zhao X Zeng W Shang L. Psychometric properties of the symptom check list 90 (SCL-90) for Chinese undergraduate students. J Ment Health. (2019) 28:213–9. 10.1080/09638237.2018.1521939 30465457

[18] Liu M Li N Cai X Feng X Wang R Xiong P. The prevalence of psychological symptoms in pregnant healthcare workers (HCWs) and pregnant non-HCWs during the early stage of Covid-19 pandemic in Chongqing, China. Front Psychiatry. (2021) 12:708698. 10.3389/fpsyt.2021.708698 34512418 PMC8424106

[19] Huang Y Wang Y Wang H Liu Z Yu X Yan J. Prevalence of mental disorders in China: a cross-sectional epidemiological study. Lancet Psychiatry. (2019) 6:211–24. 10.1016/S2215-0366(18)30511-X 30792114

[20] Riecher-Rossler A. Sex and gender differences in mental disorders. Lancet Psychiatry. (2017) 4:8–9. 10.1016/S2215-0366(16)30348-0 27856397

[21] Wei Y Li H Wang H Zhang S Sun Y. Psychological status of volunteers in a phase I clinical trial assessed by symptom checklist 90 (SCL-90) and eysenck personality questionnaire (EPQ). Med Sci Monit. (2018) 24:4968–73. 10.12659/MSM.909524 30015333 PMC6067027

[22] Zhang WR Wang K Yin L Zhao WF Xue Q Peng M. Mental health and psychosocial problems of medical health workers during the Covid-19 epidemic in China. Psychother Psychosom. (2020) 89:242–50. 10.1159/000507639 32272480 PMC7206349

[23] Zhou Y Wang W Sun Y Qian W Liu Z Wang R. The prevalence and risk factors of psychological disturbances of frontline medical staff in China under the Covid-19 epidemic: workload should be concerned. J Affect Disord. (2020) 277:510–4. 10.1016/j.jad.2020.08.059 32882508 PMC7448730

[24] Tian F Li H Tian S Yang J Shao J Tian C. Psychological symptoms of ordinary Chinese citizens based on SCL-90 during the level I emergency response to Covid-19. Psychiatry Res. (2020) 288:112992. 10.1016/j.psychres.2020.112992 32302816 PMC7151383

[25] Carr DB Ott BR. The older adult driver with cognitive impairment: “it's a very frustrating life”. JAMA. (2010) 303:1632–41. 10.1001/jama.2010.481 20424254 PMC2915446

[26] Carrey NJ. Gerald caplan, then and now: public mental health applications to the current psychological challenges of the pandemic. CMAJ. (2021) 193:E1635. 10.1503/cmaj.80232 34697099 PMC8562979

[27] Pfeifer LS Heyers K Ocklenburg S Wolf OT. Stress research during the Covid-19 pandemic and beyond. Neurosci Biobehav Rev. (2021) 131:581–96. 10.1016/j.neubiorev.2021.09.045 34599918 PMC8480136

[28] Kiely KM Brady B Byles J. Gender, mental health and aging. Maturitas. (2019) 129:76–84. 10.1016/j.maturitas.2019.09.004 31547918

[29] Seedat S Scott KM Angermeyer MC Berglund P Bromet EJ Brugha TS. Cross-national associations between gender and mental disorders in the World Health Organization world mental health surveys. Arch Gen Psychiatry. (2009) 66:785–95. 10.1001/archgenpsychiatry.2009.36 19581570 PMC2810067

[30] Goodman WK Grice DE Lapidus KA Coffey BJ. Obsessive-compulsive disorder. Psychiatr Clin North Am. (2014) 37:257–67. 10.1016/j.psc.2014.06.004 25150561

[31] Mataix-Cols D Fernandez de la Cruz L Brander G Andersson E D'Onofrio BM Ruck C. Hit-and-run: a Swedish nationwide cohort study of serious transport accidents and convictions due to traffic offenses in obsessive-compulsive disorder. Soc Psychiatry Psychiatr Epidemiol. (2021) 57:1817–27. 10.1007/s00127-021-02182-x 34779877 PMC9375758

[32] Brock H Hany M. Obsessive-Compulsive Disorder. Treasure Island, FL: Statpearls (2022). 31985955

[33] Morgan S. Mental disorders: obsessive-compulsive disorder and body dysmorphic disorder. FP Essent. (2020) 495:17–22. 32757562

[34] Miller M Azrael D Hemenway D Solop FI. 'Road rage' in Arizona: armed and dangerous. Accid Anal Prev. (2002) 34:807–14. 10.1016/S0001-4575(01)00080-X 12371785

[35] Wang X Zuo Y Jiang H Yang L. Relationship between the incidence of road traffic accidents, psychological characteristics, and genotype in bus drivers in a Chinese population. Med Sci Monit. (2018) 24:5566–72. 10.12659/MSM.909245 30096132 PMC6098670

[36] Wang L Wang Y Shi L Xu H. Analysis of risky driving behaviors among bus drivers in China: the role of enterprise management, external environment and attitudes toward traffic safety. Accid Anal Prev. (2022) 168:106589. 10.1016/j.aap.2022.106589 35151095

